# Epidemiology of subdural haemorrhage during infancy: A population-based register study

**DOI:** 10.1371/journal.pone.0206340

**Published:** 2018-10-31

**Authors:** Ulf Högberg, Jacob Andersson, Waney Squier, Göran Högberg, Vineta Fellman, Ingemar Thiblin, Knut Wester

**Affiliations:** 1 Department of Women’s and Children’s Health, Uppsala University, Uppsala, Sweden; 2 Forensic Medicine, Department of Surgical Sciences, Uppsala University, Uppsala, Sweden; 3 Formerly Department of Neuropathology, Oxford University John Radcliffe Hospital, Oxford, United Kingdom; 4 Formerly Department of Women’s and Children’s Health, Child and Adolescent Psychiatric Unit, Karolinska Institutet, Stockholm, Sweden; 5 Department of Clinical Sciences, Lund, Pediatrics, Lund University, Lund, Sweden; 6 Children’s Hospital, University of Helsinki and Folkhälsan Research Center, Helsinki, Finland; 7 Department of Clinical Medicine, University of Bergen and Department of Neurosurgery, Haukeland University Hospital, Bergen, Norway; Universidad Miguel Hernandez de Elche, SPAIN

## Abstract

**Objectives:**

To analyse subdural haemorrhage (SDH) during infancy in Sweden by incidence, SDH category, diagnostic distribution, age, co-morbidity, mortality, and maternal and perinatal risk factors; and its association with accidents and diagnosis of abuse.

**Methods:**

A Swedish population-based register study comprising infants born between 1997 and 2014, 0–1 years of age, diagnosed with SDH-diagnoses according to the (International Classification of Diseases, 10^th^ version (ICD10), retrieved from the National Patient Register and linked to the Medical Birth Register and the Death Cause Register. Outcome measures were: 1) Incidence and distribution, 2) co-morbidity, 3) fall accidents by SDH category, 4) risk factors for all SDHs in the two age groups, 0–6 and 7–365 days, and for ICD10 SDH subgroups: S06.5 (traumatic SDH), I62.0 (acute nontraumatic), SDH and abuse diagnosis.

**Results:**

Incidence of SDH was 16·5 per 100 000 infants (*n* = 306). Median age was 2·5 months. For infants older than one week, the median age was 3·5 months. Case fatality was 6·5%. Male sex was overrepresented for all SDH subgroups. Accidental falls were reported in 1/3 of the cases. One-fourth occurred within 0–6 days, having a perinatal risk profile. For infants aged 7–365 days, acute nontraumatic SDH was associated with multiple birth, preterm birth, and small-for-gestational age. Fourteen percent also had an abuse diagnosis, having increased odds of being born preterm, and being small-for-gestational age.

**Conclusions:**

The incidence was in the range previously reported. SDH among newborns was associated with difficult birth and neonatal morbidity. Acute nontraumatic SDH and SDH with abuse diagnosis had similar perinatal risk profiles. The increased odds for acute nontraumatic SDH in twins, preterm births, neonatal convulsions or small-for-gestational age indicate a perinatal vulnerability for SDH beyond 1st week of life. The association between prematurity/small-for-gestational age and abuse diagnosis is intriguing and not easily understood.

## Introduction

Subdural haemorrhage (SDH) is reported to have an incidence of 21·0–24·1 per 100 000 infants [1, 2]. Case definition depends on clinical observational studies and covers a variety of conditions, including traumatic acute subdural haemorrhage, subacute blood collections, chronic haematoma, subdural effusions, and perhaps even benign external hydrocephalus (BEH) [3] and meningitis.

SDH occurs more often in infants than in older children [4]. In a study from the UK and Ireland, comprising 186 SDH cases, Hobbs et al. found a large difference in incidence between infants (0–1 year) and children aged 1–2 years; 24·1 and 1·3 per 100 000, respectively. Further, SDH occurs more frequently in the first months than in the second half of infancy [2,5–6]. This high SDH incidence in very young infants may be due to special anatomical features in infants that increase the likelihood of developing SDH, not only caused by birth trauma, accidents, or alleged non-accidental head injuries (NAHI), but also from non-traumatic causes [7].

Current knowledge about infancy SDH is based upon hospital studies, however, only two of these used codes from the International Classification of Diseases (ICD) to retrieve cases [2, 8]. We have not been able to find any population studies on SDH that are based on ICD-10 classification. The objective of this study was therefore to retrieve data based on ICD-10 criteria from national registers and to analyse the epidemiological characteristic of SDH categorised as birth-related (P10.0), traumatic (S06.5), or acute (nontraumatic) (I62.0) in the total Swedish infant population by incidence, diagnostic distribution, gender and age distribution, co-morbidity, mortality, and maternal and perinatal risk factors; as well as the possible association of SDH with reported accidents and abuse diagnosis.

## Material and methods

**Fig 1:**
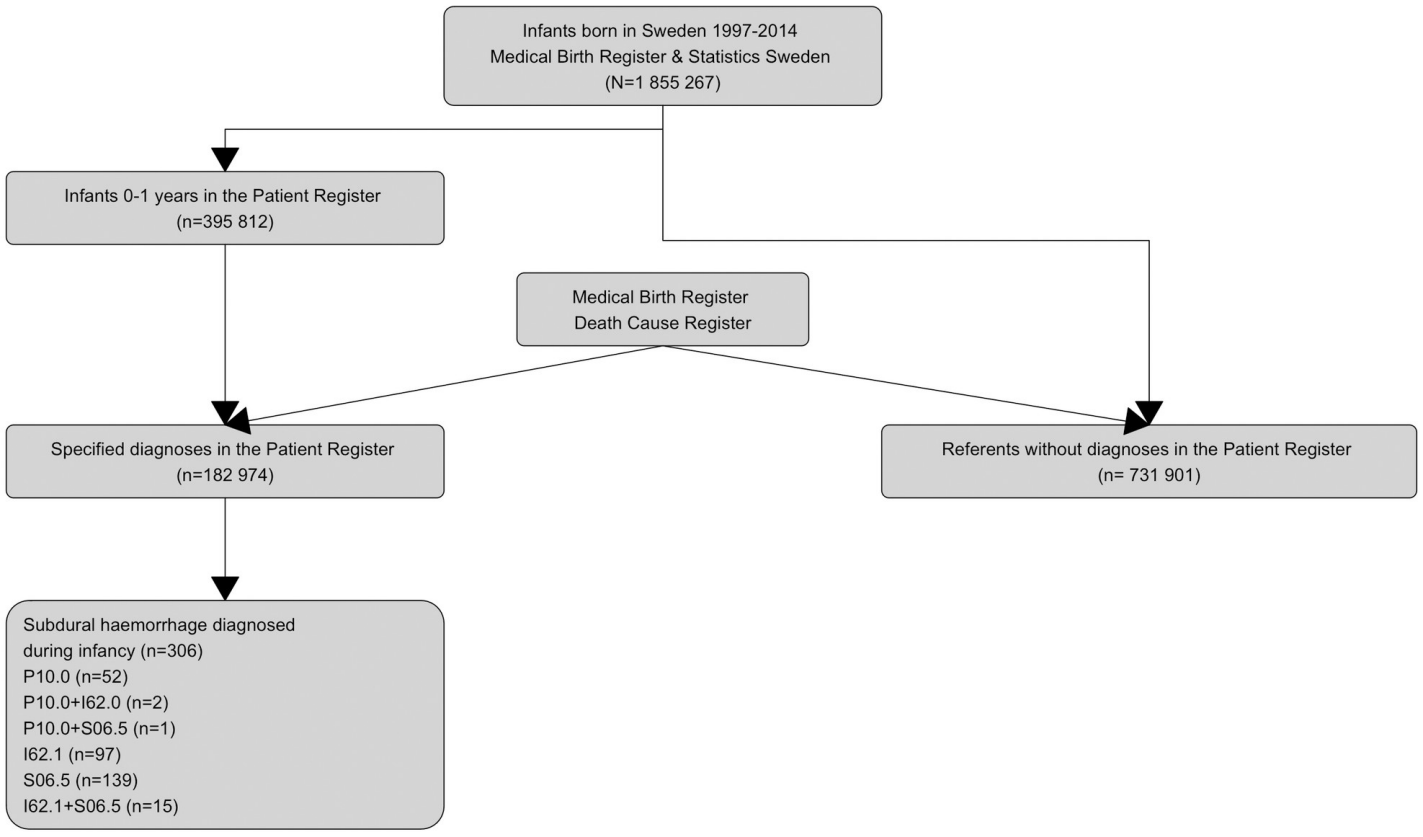
Flowchart of the study base. Source: The National Patient Register (NPR), the Swedish Medical Birth Register (SMBR), and the Death Cause Register, Swedish National Board of Health and Welfare, Statistics Sweden.

The design is a population-based register study (Fig 1). We used information from the population-based national health registers to define the study population: infants (aged 0–1 year), born in Sweden between 1997 and 2014, identified in the Patient Register [9], and admitted for hospital care (*n* = 395 812), thus having one or more diagnoses registered according to the Swedish version of the International Classification of Diagnosis (ICD-10) [10]. From those, a selection of infants with specified diagnoses were drawn as part of a larger project (*n* = 182 974) [11]. For each infant, four referents were selected, each having been born in the same year as the case and not having been included, i. e. not having received a diagnosis, in the Patient Register during the first year of life (*n* = 731 901). By using the personal identity number assigned to each Swedish resident, we linked data from the Patient Register to the Medical Birth Register [12] and Death Cause Register [13]. The final sample was 908 571 infants; this represents 49% of the total population.

For this study we included all cases of SDH according to their ICD-10 classification: SDH due to birth injury (P10.0); SDH-Acute nontraumatic subdural haemorrhage (I62.0); and SDH-Traumatic subdural haemorrhage (S06.5) (Fig 1 and S1 Table). The different ICD-codes for SDH overlapped for some cases (Fig 1). Maternal morbidity, birth complications, neonatal morbidity, transport accidents and falls, co-morbidity at time of diagnosis, and abuse diagnosis (observation for suspected abuse, battered baby syndrome, and maltreatment syndrome), were defined according to ICD-10 (S1 Table). Maternal age and parity, sex, multiple births, gestational week (≤31; 32–36; 37+ weeks), small-for-gestational age (<2·5 or <10 percentiles), and birth asphyxia (Apgar score <4 at 1 min, <4 at 5 min, <4 at 10 min) were registered. Mode of delivery was categorized as: 1. elective caesarean, 2. normal vaginal delivery (spontaneous start of labour, only vertex presentation), 3. not normal spontaneous vaginal delivery (induced labour, dystocic labour, brow or breech, and others), 4. emergency caesarean, and 5. assisted vaginal delivery (vacuum or forceps).

We determined:

- Incidence of all included SDH and of the two subgroups S06.5 and I62.0, in addition to SDH combined with abuse diagnosis.
- Risk factors for SDH 0–6 days, all SDH 7–365 days, then separately for the subgroups S06.5, I62.0, for those infants who had both diagnoses (S06.5 and I62.0), and, finally, for SDHs combined with abuse diagnosis at 0–365 days.
- Co-morbidity for all SDHs except birth-related, only S06.5 and only I62.0.

Population incidences were calculated as cases per 100 000. Mantel-Haenszel Chi-Square or Fisher´s exact tests were used to assess differences in non-parametric variables, means and analysis of variance (ANOVA). Crude and adjusted odds ratio (OR) analyses with 95% confidence intervals were applied to assess possible associations between maternal and perinatal exposures and the outcome. The statistical software package IBM SPSS 25·0 (IBM Corp., Armonk, IL) was used for data analyses.

The Regional Ethical Review Board in Uppsala approved the study (2014-11-19 No 383).

## Results

In all, 306 cases of infant SDH were found during the years 1997–2014, which gives an overall incidence of 16·5 per 100 000 infants. The distribution by diagnosis is shown in Fig 1: 55 (18.0%) had P10.0 (birth injury), 139 (45.3%) had S06.5 (traumatic subdural haemorrhage), and 97 (31.7%) had I62.0 (acute nontraumatic subdural haemorrhage), while 15 (4.9%) had both diagnoses (S06.5 and I62.0).

When split between the diagnostic subgroups, the incidence for each of these was 7·5 for I62.0, 5·2 for S06.5, 3·0 for P10.0, and 0·8 for the combination of I62.0 and S06.5.

A total of 43 (14%) had a combination of abuse and SDH diagnoses; of these, 19 had S06.5, 15 had I62.0, and nine had both. The incidence of SDH with abuse diagnosis was 2·3 per 100 000 infants.

### Mortality

Case-fatality rate was 6·2% (19/306). The causes of death were: malformations (5 infants), perinatal causes (1 infant), Menkes disease (1 infant), leukaemia (1 infant), subarachnoid haemorrhage (1 infant), unspecified acute lower respiratory infection (1 infant), peritonitis (1 infant), accidents (3 infants), specified accident events undetermined intent (3 infants), abuse/homicide (2 infants). Concomitant diagnoses were: intracerebral haemorrhage, traumatic brain oedema, cerebral infarction, skull fracture, sepsis, heart disease, cardiac arrest, and coagulopathy (S1 Table).

Of the five infants who died from accidents or abuse/homicide, two died at the age of 38 and 355 days without any diagnosis registered prior to death. For the other three, the following diagnoses had been registered prior to death: anoxic brain injury due to drowning after a boat accident, unspecified anaemia with failure to thrive, and extremely low birth weight in combination with acute pharyngo-laryngitis and severe anaemia.

### Gender distribution

**Fig 2:**
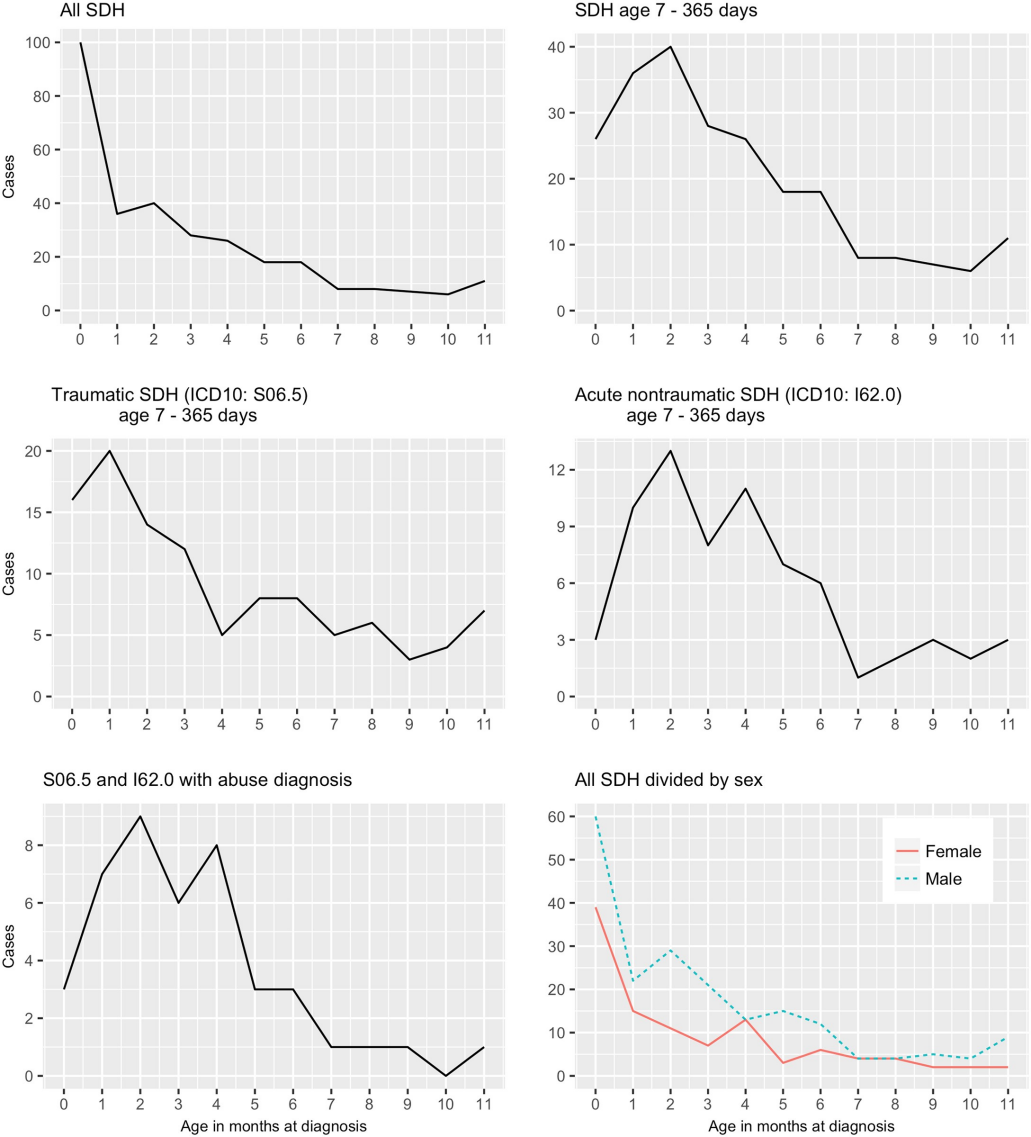
Cases of subdural haemorrhage (SDH) during infancy by month of diagnosis; All SDH, SDH age 7–365 days, Traumatic SDH S06.5 age 7–365 days, SDH (acute (nontraumatic I62.0 7–365 days, S06.5, and I62.0 with abuse diagnosis, All SDH divided by sex.

Compared with the general population, there was a marked male preponderance. Of all 306 infants, 198 were boys (64·7%) and 108 were girls (35·3%) (*p* < .001). This gender preponderance was also present and stable when the material was split into diagnostic SDH subgroups (P10.0, S06.5, and I62.0) (S2 Table) and months with exception of months 4^th^, 7^th^, 8^th^ (Fig 2); or between the trauma mechanisms falls and transport accidents. In this regard, there was no statistically significant difference in proportions between the diagnostic subgroups.

### Age at diagnosis

One-fourth of the cases, 74 (24%), occurred during the first week of life, 40 (13%) occurred after the age of 6 months. Mean and median age at diagnosis for all SDH cases was 3·3 and 2·5 months, respectively (S2 Table). For the 232 infants older than one week (7–365 days), the mean and median age was 4·3 and 3·5 months, respectively. When excluding the first week of life, there is a marked peak at two months of age (Fig 2). Most SDH cases were diagnosed during the first 6 months of life; only 40 (17·2%) of these 232 infants were older at diagnosis. There was no statistically significant differences (ANOVA) in mean and median age between the diagnostic subgroups for birth-related SDH, S06.5, and I62.0 (S2 Table).

### Trauma mechanisms

**Fig 3:**
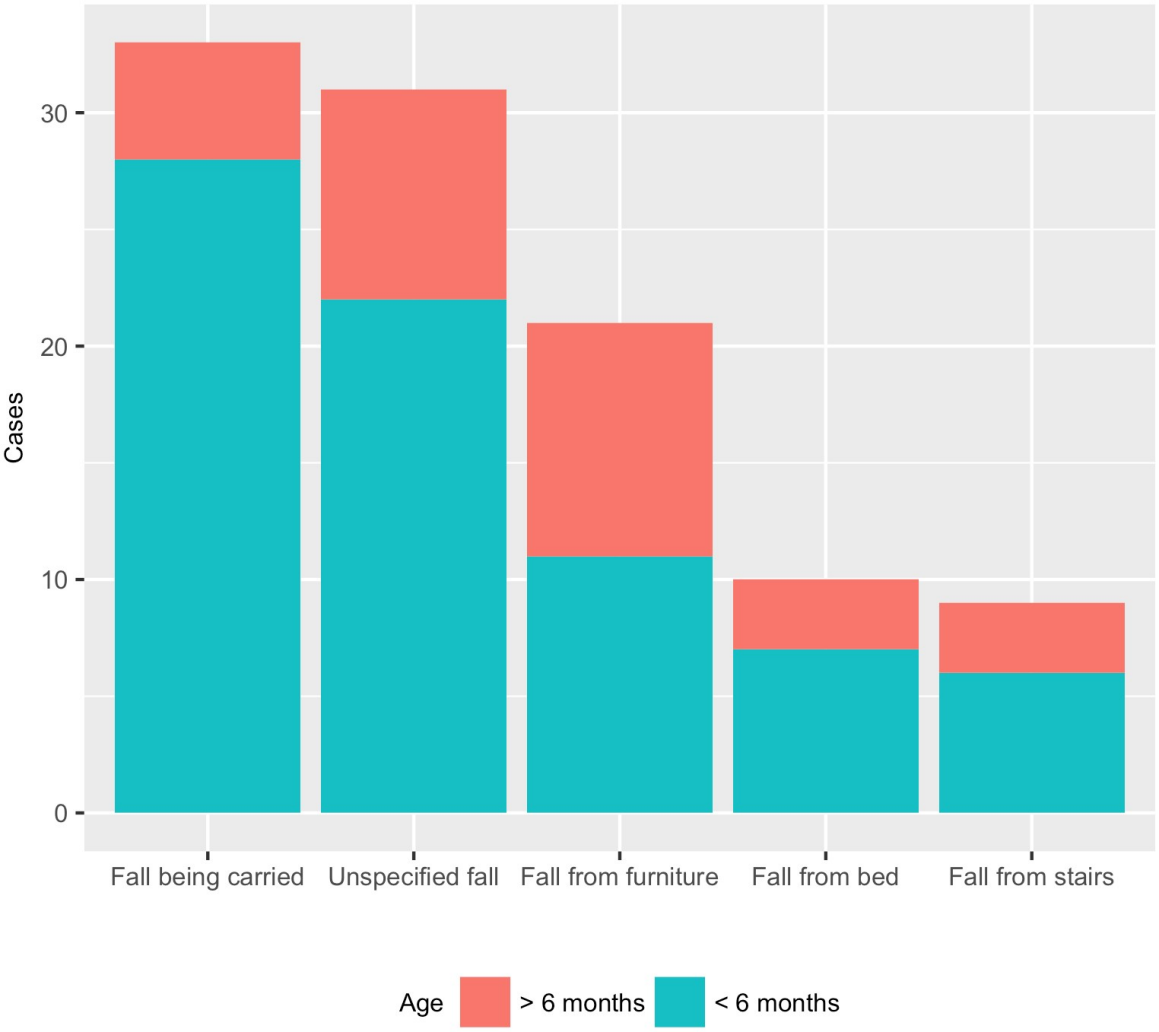
Subdural haemorrhage with reported fall accident at time of diagnosis (*n* = 104) by fall accidents (1 case missing) and age for infants born between 1997 and 2014 in Sweden.

An accidental fall at time of diagnosis was reported for 104 infants (34·0%), 67 boys (64·4%) and 37 girls (35·6%), with a mean age of 122.7 days; nine occurred during first week of life. The distribution of the fall cases by age and type of fall are shown in Fig 3. The majority– 74 (71·2%) of these falls–occurred during the first 6 months of life. Falls while being carried, from bed or stair, and unspecified falls were more commonly reported during the first six months, whereas falls from furniture were more equally distributed throughout the first year of life. Seven out of 43 abuse cases also had accidental fall reported.

A transport accident was reported at time of diagnosis for 39 infants (12·7%), with a mean age of 3·3 months. Even among the transport accidents, there was a marked male preponderance: 25 (64·1%) boys, and 14 (35·9%) girls. One infant with SDH and abuse diagnosis had a transport accident 1 day before diagnosis.

### Co-morbidity

Relatively few infants had a co-morbidity diagnosis. Table 1 shows the co-morbidity diagnoses among the non-birth related SDH-cases, including the subgroups S06.5 and I62.0. Before or at diagnosis, 26 (10·4%) had severe infections: sepsis, pneumonia, or meningitis; 18 (7·2%) had a diagnosis of hydrocephalus. Vomiting and gastro-oesophageal reflux disease was relatively rarely reported. Before an SDH-diagnosis, five children were reported to have had infant colic; none of those had an abuse diagnosis. Predominant at diagnosis were skull fractures– 71 (28·2%)–most of these had a traumatic SDH-diagnosis (< *p* .001); the second-most common diagnosis was convulsions– 31 (12·4%)–without any differences between the diagnostic subgroups. Retinal haemorrhage was diagnosed in 27 (10·8%) infants, of these infants, I62.0 was more common than S06.5 (< *p* .05), Eighteen of these infants also had an abuse diagnosis (Table 1). Thirty-eight of the 43 cases with an abuse diagnosis also had long bone fractures, and 7 had rib fractures.

**Table 1:** Co-morbidity before and at time of diagnosis (± 7 days) of subdural haemorrhage (S06.5), traumatic subdural haemorrhage (S06.5), acute (nontraumatic) subdural haemorrhage (I6.20) among infants born in Sweden 1997–2014. Source: The National Patient Register.

Diagnoses^3^	*N*^4^	All SDH^1^ (*n* = 251)		Traumatic SDH (*n* = 139)		Acute (non-traumatic) SDH^2^ (*n* = 112)	
		Before diagnosis	At diagnosis	Before diagnosis	At diagnosis	Before diagnosis	At diagnosis
Sepsis, meningitis, pneumonia	19 591	15	11	5	1	10	10^b^
Hydrocefalus	1 094	11	7	4	2	7	5
Skull fracture	1 521	5	71	4	64^a^	1	7
Brain contusion	9 097	3	11	3	11	0	0
Anoxic brain injury, brain oedema, stroke	541	1	11	1	2	0	9
Convulsions	13 174	6	31	1	13	5	18
Retinal haemorrhage	147	0	27	0	10	0	17^c^
Vomiting	21 436	5	4	0	0	5	4
GERD^5^	4 242	1	2	0	0	1	2
Failure to thrive	10 415	2	0	0	0	2	0

^1^P10.0 excluded,

^2^Including also those having both diagnoses (I620 + S0650) (*n* = 15),

^3^No cases of Apparent life-threatening event in infant (ALTE) or sinus venous thrombosis (SVT) among the SDH-cases,

^4^*N* = 908,565,

^5^Gastro-esophageal reflux disease (GERD)

Mantel-Haenszel Chi-Square or Fisher exact *p*-value comparing traumatic and acute (non-traumatic) SDH (a <0.001, b<0.01, c <0.05).

### Maternal and perinatal risk factors

Maternal and perinatal characteristics, age at diagnosis, and SDH subgroups are shown in S2 Table, Tables 2 and 3. When comparing all SDH cases with the general population, statistically significant increases were found for the following factors: preeclampsia, dystocic labour, non-normal mode of delivery, prematurity, multiple birth, small-for-gestational age, birth asphyxia, cephalic haematoma, respiratory distress, neonatal sepsis, convulsions, and other cerebral disturbances of the newborn (S2 Table). Both infants with SDH and controls without SDH had a male preponderance for neonatal morbidity diagnoses, but for those with SDH, it was more pronounced when they had cephalic haematoma (males 71·4%), or diagnosis of other cerebral disturbances of the newborn (80% males—data not shown). For the maternal and perinatal risk factors, no statistically significant differences could be found between S06.5 and I62.0, or between any of those two and abuse diagnosis (S2 Table). When dichotomized between high and low age (below or above 34 years), maternal age did not seem to be of significance.

**Table 2:** Risk factors for infants diagnosed with subdural haemorrhage (SDH), by category traumatic SDH, acute (nontraumatic) SDH, and SDH concomitant with abuse diagnosis, and by age 0–6 and 7–365 days during the years 1997–2014 in Sweden (Swedish National Board of Health and Welfare: Patient Register, Medical Birth Register). Crude odds ratios (OR) and 95% confidence intervals.

Maternal and perinatal risk conditions		SDH 0–6 days	SDH 7–365 days			SDH & abuse diagnosis 0–365 days
		(*n* = 74)^1^	All (*n* = 232)	Only traumatic SDH (*n* = 108)^2^	Only acute (nontraumatic) SDH (*n* = 69)^3^	(*n* = 43)^4^
		OR (95% CI)	OR (95% CI)	OR (95% CI)	OR (95% CI)	OR (95% CI)
**Preeclampsia**		**2·34 (1·07–5·10**)	**2·11 (1·34–3·34**)	1·09 (0·44–2·67)	2·13 (0·92–4·93)	**3·63 (1·53–8·61**)
**Dystocic labour**		**2·74 (1·68–4·46**)	0·98 (0·68–1·41)	1·30 (0·80–2·11)	0·54 (0·24–1·46)	1·11 (0·49–2·49)
**Mode of delivery** **(Ref: Planned Caeserean)**	**Normal vaginal delivery**	1·67 (0·39–7·15)	0·51 (0·35–0·73)	1·00 (0·51–1·96)	0·49 (0·25–0·96)	0·40 (0·17–0·91)
	**Else spontaneous vaginal delivery**	3·02 (0·69–13·3)	0·56 (0·36–0·86)	0·91 (0·43–1·92)	0·63 (0·29–1·35)	0·49 (0·19–1·26)
	**Emergency caesarean**	4·18 (0·89–19·7)	0·62 (0·37–1·05)	0·94 (0·38–2·31)	0·76 (0·31–1·89)	0·65 (0·21–2·0)
	**Asssisted vaginal delivery**	**18·8 (4·50–78·8)**	0·44 (0·23–0·83	1·00 (0·39–2·54)	0·23 (0·05–1·03)	0·32 (0·07–1·48)
**Sex** **(Ref: females**	**Male**	1·54 (0·96–2·46)	**1·78 (1·36–2·33)**	**1·53 (1·04–2·26)**	**2·29 (1·36–3·85)**	1·75 (0·93–3·27)
**Multiple birth**		1·89 (0·69–5·18)	**2·78 (1·72–4**·50)	1·95 (0·85–4·43)	**3·74 (1·71–8·16)**	**3·39 (1·21–9·50)**
**Gestational week** **(Ref:37+)**	**32**–**36**	1·07 (0.39–2.94)	**2·66 (1·80–3·93)**	**2·08 (1·11–3·88**)	**2·79 (1·38**–5·63)	1·07 (0·39–2·94)
	**<32**	**4·17 (1·31–13·3)**	**3·33 (1·57–7·07)**	1·96 (0·48–7·95)	3·21 (0·78–13·2)	**4·17 (1·31–13·3)**
**Small-for-gestational-age**	**<2.5th percentile**	1·87 (0·59–5·95)	**2·52 (1·41–4·51)**	1·29 (0·41–4·08)	**2·93 (1·06–8**·08)	**6·14 (2·40–15·7)**
	**<10th percentile**	0·91 (0·42–1·99)	**1·46 (1·01–2·11)**	1·31 (0·75–2·29)	1·69 (0·88–3·22)	1·72 (0·76–3·87)
**Birth Asphyxia**	**Apgar <4 1 minute**	**20·4 (11·7–35·5)**	1·63 (0·67–3·94)	1·39 (0·34–5·64)	1·09 (0·15–7·82)	-
	**Apgar <4 5 minutes**	**34·2 (14·8–78·9)**	-	-	-	-
	**Apgar <4 10 minutes**	**50·5 (20·4–125)**	-	-	-	-
**Neonatal diagnosis**	**Birth injury to the scalp**	**22·9 (12·8–41·1)**	**3·06 (1·44–6·4**8)	2·81 (0·89–8·84)	2·93 (0·72–12·0)	-
	**Birth injury to the skeleton**	**21·7 (14·4–45·3)**	-	-	-	-
	**Respiratory distress**	**3·55 (1·77–7·13)**	**1·90 (1·14–3·16)**	1·78 (0·83–3·82)	1·58 (0·58–4·33)	1·92 (0·60–6·21)
	**Sepsis**	**4·12 (1·30–13·1)**	**3·03 (1·43–6·43)**	2·78 (0·88–8·77)	2·91 (0·71–11·9)	2·32 (0·31–16·5)
	**Convulsions**	**271 (170–431)**	**11·6 (5·46–24·7)**	3·48 (0·49–24·9)	**16·9 (5·32–53·9)**	**8·86 (1·22–64·4**)
	**Other cerebral disturbances of the newborn**	**117 (50·9–272)**	-	-	-	-

^1^33 cases had traumatic SDH, 20 cases had non-traumatic SDH,

^2^16 cases excluded that had both traumatic and non-traumatic SDH, 19 cases with abuse diagnosis excluded

^3^16 cases excluded that had both traumatic and non-traumatic SDH, 14 cases with abuse diagnosis excluded,

^4^ 19 had traumatic SDH, 15 had non-traumatic SDH and 9 had both

**Table 3:** Adjusted odds ratios (AOR) and 95% confidence intervals for infants diagnosed with subdural haemorrhage (SDH) at age 7–365 days by category acute (nontraumatic) SDH and SDH concomitant abuse diagnosis (observation for suspected abuse, battered baby syndrome, maltreatment syndrome) during the years 1997–2014 in Sweden (Swedish National Board of Health and Welfare: Patient Register, Medical Birth Register).

Maternal and perinatal risk conditions		SDH 7–365 days (*n* = 232)			Only acute (non-traumatic) SDH 7–365 days (*n* = 69)			SDH & abuse diagnosis 0–365 days (*n* = 43)		
		Model 1^1^	Model 2^2^	Model 3^3^	Model 1^1^	Model 2^2^	Model 3^3^	Model 1^1^	Model 2^2^	Model 3^3^
		AOR (95%CI)	AOR (95%CI)	AOR (95%CI)	AOR (95%CI)	AOR (95%CI)	AOR (95%CI)	AOR (95%CI)	AOR (95%CI)	AOR (95%CI)
**Preeclampsia**		**2·17 (1·37–3·45)**	**1·82 (1·08–3·07)**	1.52 (0·88–2·61)	2·26 (0·97–5·25)	1·55 (0·55–4·40)	1·25 (0·43-·3·69)	**3·66 (1·53–8.7**5)	2·68 (0·99–7·17)	2·14 (0·75–6·13)
**Sex** **(Ref: females)**	**Male**	**1·78 (1·36–2·33)**	**1·78 (1·33–2·35)**	**1·77 (1·35–2·32)**	**2·29 (1·36–3·86)**	**1·77 (1·33–2·35)**	**2·22 (1·28–3·49)**	1·75 (0·93–3·27)	1·87 (0·96–3·65)	1·86 (0·96–3·62)
**Multiple birth**		**2·85 (1·75–4·63)**	**2·85 (1·75–4·63)**	**1·86 (1·09–3·17)**	**3·69 (1.68–8.12)**	**2.85 (1.75–4.63)**	**2.53 (1.04–6.10)**	**3.86 (1.36–10.9)**	-	2.13 (0.66–6.83)
**Gestational week** **(Ref:37+)**	**32–36**	**2.66 (1.80–3.93)**	**2.39 (1.51–3.78)**	**2.39 (1.51–3.78**	**2.80 (1·39–5·65)**	**2·36 (1·01–5·56)**	**2·36 (1·01–5·56)**	**2·57 (1·01–6·57)**	2·23 (0·77–6·40)	2·23 (0·77–6·40)
	**<32**	**3·32 (1·57–7·06)**	**3·50 (1·51–8·11)**	**3·50 (1·51–8·11)**	3·21 (0·78–13·1)	3·91 (0·9–16·9)	3·91 (0·9–16·9)	**8·07 (2·48–28·3)**	**4·60 (1·01–20·9)**	**4·60 (1.01–20.9)**
**Small-for-gestational-age**	**<2.5th percentile**	**2·59 (1·44–4·64)**	**2·61 (1·45–4·68)**	**2·01 (1·09–3·68)**	**3·13 (1·13–8·68)**	**3·13 (1·13–8·6**8)	2·39 (0·82–6·92)	**6·06 (2·35–15·6)**	**6·06 (2·35–15·6)**	**4·57 (1·66–12·5)**
**Neonatal diagnosis**	**Birth injury to the scalp**	**2·91 (1·37–6·20)**	**3·17 (1·49–6·76)**	**3·33 (1·56–7·09)**	2·86 (0·70–11·7)	3·19 (0·78–13·2)	3·36 (0·81–13·5)	-	-	-
	**Respiratory distress**	**1·84 (1·11–3·07)**	1·59 (0·88–2·56)	1·02 (0·56–1·88)	1·52 (0·55–4·18)	1·33 (0·41–4·3)	0·93(0·46–1·86)	1·88 (0·58–6·08)	1·22 (0·29–5·13)	0·55 (0·12–2·53)
	**Sepsis**	**2·94 (1·39–6·25)**	**3·28 (1·53–7·01)**	1·99 (0·86–4·57)	2·84 (0·69–11·6)	3·19 (0·77–13·2)	1·92 (0·41–9·11)	2·20 (0·30–16·0)	2·01 (0·28–15·2)	0·79 (0·90–6·74)
	**Convulsions**	**11·5 (5·43–24·5)**	**10·8 (4·77–24·4)**	**9·51 (4·19–21·6)**	**17·0 (5·35–54·3)**	**12·5 (3·03–51·4)**	**10·8 (2·60–45·3)**	**8·67 (1·19–63·0)**	**8·51 (1·16–62·5)**	**7·31 (0·99–54·2)**

^**1**^Model 1: adjustment for parity and maternal age, and child sex,

^**2**^Model 2: adjustment for parity and maternal age, child sex, small-for-gestational age, multiple birth,

^**3**^Model 3: adjustment for parity and maternal age, and child sex, small-for-gestational age, multiple birth, gestational week

SDH (0–6 days) had distinct characteristics associated with dystocic labour [OR 2·74 (95% CI 1·68–4·46)], emergency caesarean delivery, assisted vaginal delivery [OR 18·8 (95%CI 4·60–78·8)], asphyxia, preterm birth, birth injury to the scalp or skeleton, and neonatal morbidity, especially convulsions [OR 271 (95%CI 171–431)] and other cerebral disturbances of the newborn [OR 117 (95% CI 50·9–272)] (Table 2). Mode of delivery had no association with SDH beyond 6 days of age. Male sex and preterm birth (32–36 weeks) had increased odds for S06.5, but had no other risk increase.

Adjusted odds ratios (aOR) are shown in Table 3. Multiple birth, preterm birth, small-for-gestational age (< 2.5 percentile), and neonatal convulsions showed increased odds for both I62.0 and abuse-related SDH. For isolated diagnoses of abuse-related SDH, the odds were even higher than for I62.0: being born before week 32 [aOR 4·6 (95% CI 1·01–20·9)], or being small-for-gestational age [aOR 4·57 (95%CI 1·66–12·5)] (Table 3).

## Discussion principal findings

This national, ICD-10-based study comprising 306 infants shows an incidence of infant SDH of 16·5 per 100 000 infants. One-fourth occurred during the first week of life; these cases had distinct birth risk profiles of difficult birth, preterm birth, and neonatal morbidity. Male sex, multiple birth, preterm birth, small-for-gestational age, and neonatal convulsions had increased odds for SDH at 7–365 days, especially for acute nontraumatic SDH—I62.0. Light or moderate traumas (fall accidents) were reported in 1/3 of the cases. One in seven– 14%–of all SDH cases had a concomitant diagnosis of abuse; these had, surprisingly, an even higher perinatal risk profile than the I62.0 subgroup, with a strong association between prematurity/small-for-gestational age and abuse diagnosis; moreover, those with abuse diagnosis also had a higher number of diagnoses of retinal haemorrhage, rib fractures, and long bone fractures.

### Gender distribution

Throughout the entire set of material, there was a marked male preponderance, indicating that infant boys somehow are twice as prone to encounter a SDH as girls are, regardless of the diagnostic or age subgrouping of the cases. Boys have previously been shown to be overrepresented in very early publications on infant SDHs [4, 5]. Similar gender preponderance has previously been described for several intracranial conditions, such as Benign External Hydrocephalus—BEH [14, 15]–also known as “Benign Enlargement of the Subarachnoid Spaces”–BESS—or “macrocephaly”, or many other terms. The underlying mechanism for this gender difference remains obscure, but the possibility exists that, in at least some of these reported cases, there is a common pre-existing condition that occurs more frequently in males than in females, and that renders boys more susceptible to intracranial bleeding, even from minor traumas or no trauma at all. BEH is such a condition [16–23]. In a recent article on the association between BEH/BESS and subdural collections, Tucker et al. observed “that greater depth of the subarachnoid space is associated with increased prevalence of such collections” [24]. As previously known, boys are overrepresented in neonatal morbidity, as well as for vascular instability. In males, lung maturation is delayed compared to females, and male newborn infants have higher incidences of birth asphyxia as well as common neonatal disorders linked to oxidative stress. It has been suggested that the lower antioxidant capacity in males results in increased risk of pulmonary vasoconstriction, vascular instability, and hypoxia [25]. These constitutional differences between boys and girls might explain some of the preponderance of SDH in boys.

In this context, it is of interest that, in 1953, Guthkelch had already discussed the possibility that the male SDH preponderance could be explained by a birth trauma, stating: “the preponderance of males might be explained on the basis that male infants have bigger heads than females, and therefore more likely to sustain intracranial injury at birth.” [5] This hypothesis is further supported by recent interpretations by Squier, Vinchon, and Miller [15, 21, 26].

### Age distribution

In 1944, Ingraham and Matson had already observed in their pioneering article that SDH in infants mainly occurred during the first months of infancy [4]. We found a similar age distribution in our material; only 13% of the infants were diagnosed with an SDH after the age of 6 months. A skewed age distribution towards the first months in infancy has been demonstrated by several authors [2, 6, 27–29].

### Trauma mechanisms and other possible causes of SDH

Subdural blood collections, most asymptomatic, occur in vaginal birth in very high percentages [30–33]; almost all resolve without symptoms, but few cases may remain and might develop into larger haematomas or hygromas [4, 5, 32, 33].

Numerous reports have demonstrated that BEH in infants may predispose for spontaneous, nontraumatic subdural bleedings [17–24, 27, 34–38], and subdural effusions in BEH might also be interpreted as SDH [39]. Subdural haemorrhage may also be secondary to sinus venous thrombosis [40].

Even for the SDH with a traumatic aetiology, and for transport accidents analysed separately, there was a marked male preponderance. For infants, one would expect such incidents to be equally distributed among the genders; when it is not, one may wonder whether there is some underlying biological factor that renders boys more vulnerable to even trivial external trauma, as discussed above.

### Co-morbidity

In our study, very few co-morbidity diagnoses were registered, whereas clinical studies have reported much higher proportions of convulsions, vomiting, and irritability [1, 4, 6]; one reason could be that our data are restricted to registers and are not from journal audits. It is possible that such symptoms are less likely to be included in the type of diagnosis register we have used for the retrieval of patients.

### Perinatal risk factors

Prematurity and small-for-gestational age are distinct findings of increased risk of SDH-Acute non-traumatic and SDH with abuse diagnosis. Few studies have addressed these associations. Prematurity has been reported for SDH with abuse diagnosis by Keenan et al. [41], but not by Hobbs et al. [1] Infants born preterm are more susceptible to oxidative stress during the foetal-to-neonatal transition [42]; we speculate that this might be one mechanism behind our results.

### Strengths and limitations

One strength of the present study is that the data were retrieved from national registers, thus covering the entire country and, consequently, we included all infants who were given an SDH diagnosis throughout the study period. Another strength is that the same ICD-10 classification was used during the entire study period. The dataset including the referents contained 49% of all children born in Sweden during the study period, strengthening its population representativeness. Regarding exposure, a biological association is strengthened by the gradient of odds for SDH shown for preterm birth and small-for-gestational age. The validity of the Swedish health registers is considered to be high, both in regards to the Medical Birth Register [12] and the Patient Register [43], although the specific diagnoses used in this study have not been validated.

This lack of validation for the SDH diagnoses is a limitation, as we had no access to detailed clinical documentation on each patient’s history, condition, neuroimaging, ophthalmological examinations, or treatment. Neither could we assess the initiation time point of the SDH. In addition, we have no validation of the registered diagnoses of co-morbidity, as we have had to rely completely on the hospital physicians’ choice of ICD-10 classifications. This is a major clinical dilemma, as there are no available SDH diagnoses in ICD-10 for common clinical entities, such as “chronic subdural haematoma”, “subacute blood collections”, “subdural hygroma”, or “subdural effusion” [3], or any of the other conditions that, in the present ICD version, now have to be allocated into one of the available SDH diagnoses (I62.0 or S06.5). Thus, the ICD-10 does not allow differentiations that are required for sufficient study quality, such as between acute, subacute, or chronic subdural blood/fluid collections.

Another weakness relates to the registering of traumatic SDHs. We know that parents suspected of violent shaking or intended head injury can be asked to give an explanation for an SDH that is assumed to be of traumatic origin; in that situation, they may give emphasis to minor traumas during the preceding days, such as bumping the head into a bed-post or a short fall onto a sofa, as reported in the UK and Ireland in 41/97 of the SDH cases diagnosed as abuse [1], while we had a corresponding 7/43. If the caregivers gave this information to the clinician in charge of classification, it is possible that the SDH in those cases would be registered as being traumatic with a corresponding ICD-10 classification of fall accidents (W01-19), however, the different proportions between the UK and Ireland and Sweden might also reflect a different understanding of SDH cause mechanisms. One limitation of this epidemiological study is that the design cannot ascertain causality; it can only suggest risk associations. Hypothetical inferences were only reported where statistically significant associations are found. Correction for increased risk of type I error by multiple testing has not been performed, as in the final model a limited number of exposures where applied to outcome subcategories. A major limitation is that subtle neonatal SDH was likely undetected in this study design, as there is no routine head scan of all newborns in Swedish health care. Furthermore, the ICD-10 criteria for diagnosis of abuse are not explicitly stated.

### Previous research

The SDH incidence found in this study was in the range previously reported, although lower than that reported in a study from the British Isles over a one-year data collection period in 1998–99 [1].

The case fatality rate in our study was lower; 6·2%, compared to the 19% reported by Hobbs et al. in 1998–1999 [1]. One interpretation might be that the data are retrieved from different populations and there may be a selection bias towards more serious cases being reported to the British Paediatric surveillance unit over that one year. Our study was performed up to 15 years after Hobbs et al., when imaging techniques are more readily available, thus allowing the detection of less severe conditions.

Our study identified 18% birth-related SDH, a finding similar to the 14% reported by Hobbs et al. [1], but this is lower than that reported in 1944 by Ingraham and Matson– 29% [4]. The present study supports the hypothesis that, even in modern obstetric practice, a traumatic birth and severe neonatal morbidity increases the risk of SDH in neonates within, but not beyond, the first week of life. However, the distinct, increased odds of being twin, being born premature, having neonatal convulsions, or being small-for-gestational age, when diagnosed with acute non-traumatic SDH, do indicate a perinatal vulnerability for SDH beyond the first week of life. As our study cannot ascertain a causal relation, the enhanced odds only apply to the risk of receiving an SDH diagnosis or a diagnosis of abuse. It might be speculated that later onset cases are babies who develop a hygroma with a risk of re-bleeding [1, 3, 44].

Fall accidents were reported for 104 infants, all classified as traumatic. In the British Isles, only 7 out of 189 cases were classified as accidents, although carers provided a history of minor trauma in 41 of the 97 non-accidental head injury (NAHI) cases [1]. The specified fall accidents in our study involved falls classified as a slight or moderate trauma according to Landin´s Modified Trauma Levels [45], which are reported by the parents in cases of SDH [46]. In a case study of traumatic head injury among children aged below 3 years, 45% of the infants with SDH had a reported fall, with 2/3 of these from less than 80 cm [47]. Studies on infants, who have died after short falls, and biomedical models, indicate that a fall from as low as 0.3 m might generate forces equal to 100 G [48, 49].

In our study, one in seven cases had a diagnosis of abuse, 2·3 per 100 000, while more than half in the UK and Republic of Ireland were classified as NAHI, at 14·2 per 100 000 [1], i.e., a 6-fold difference. Furthermore, a 10-fold difference was found between our study and a Scottish study of shaken impact syndrome, with 24·6 per 100 000 [50]. This difference between Sweden and the UK seems to be consistent with what was reported as “maltreatment syndrome or assaults” by Gilbert et al [51] (their fig 3) for infants, while for children aged 1 year, this difference is small. This indicates a higher risk of receiving an abuse diagnosis in UK than in Sweden. A significant result from our study is the close similarities between acute nontraumatic SDH and SDH with abuse diagnosis in perinatal risk profiles, such as preterm birth, small-for-gestational age, and multiple births. This difference might also reflect dissimilar diagnostic considerations in Sweden and the UK and Republic of Ireland, and this may be one reason for the 6-fold differences in SDH and abuse diagnosis [1] however, a recent study shows that, in Sweden, SDH is also strongly associated with an abuse diagnosis [11].

### Possible implications

The present study reflects the shortcomings of the ICD-10 system in classifying SDH; there is no code for chronic or subacute haematomas, nor is there a code for subdural fluid collections containing small amounts of blood or no blood at all, or what in clinical terms are described as “hygromas”. In the present ICD version, any of these chronic conditions therefore must be categorized into one of the two available SDH diagnoses (I62.0 or S06.5), both indicating that the SDH was caused by an acute internal or external event. In the diagnostic work-up of suspected violent shaking, this lack of diagnostic differentiation may represent a serious pitfall, as even obviously chronic subdural collections have to be classified as an acutely acquired condition.

The vulnerability of boys in attaining SDH, whether traumatic or not in the first months of life, is indeed intriguing and may reflect the possibility that at least some of these male infants are predisposed to subdural blood collections with no or only minor trauma. External hydrocephalus is such a condition; it occurs mainly in boys and during the first months of life, and it predisposes for subdural fluid collections. The fact that perinatal risk factors, such as being prematurely born or being small-for-gestational age, are highly associated with an SDH-related abuse diagnosis underscores the suggestion that the diagnostic work-up should be cautious and should include consideration about whether external hydrocephalus might be an underlying cause of the SDH.

As no routine head scan is performed on all newborn infants, there is a high risk that subtle SDH related to perinatal risk factors will not be detected postnatally. For this reason, a future investigation of the long-term development of infants with postnatal subdural blood, using non-radiation head scans, is warranted.

This study cannot fully assess whether there has been an over-diagnosis of shaken baby syndrome or abusive head trauma; however, the similar risk profiles for infants with SDH and infants with SDH and abuse diagnosis indicate that the diagnostic process might be uncertain. Thus, the insufficient scientific evidence of the triad in identifying traumatic shaking, as shown in a systematic literature review of the Swedish Agency for Health Technology Assessment and Assessment of Social Service [52], raises serious medico-ethical concerns.

This population study should be followed by a clinical study with access to information from clinical records, patient history, and imaging, such as MRI scans.

## Conclusion

The total incidence of SDH was in the range previously reported– 16·5 per 100 000 infants—with the majority of cases occurring during the first months of life (median 2·6). Boys were twice as prone to having an SDH-diagnosis across all ages and SDH subgroups, indicating an increased vulnerability to minor trauma and/or increased bleeding sensitivity in male infants. SDH among neonates was associated with birth and neonatal morbidity. Traumatic SDH during infancy was strongly related to fall accidents. Acute nontraumatic SDH and SDH with abuse diagnosis during infancy had similar perinatal risk profiles. To be able to correctly identify all causes of intracranial bleeding, population-based newborn cohorts should undergo repeated imaging studies from birth to several months of age.

## Supporting information

S1 Table Definitions of subdural haemorrhage diagnosis, co-morbidity, neonatal morbidity and accidents. Swedish version of 10^th^ revision of the International Statistical Classification of Diseases (ICD-10).
(DOCX)

S2 Table Mean and median days of SDH diagnosis, distribution of maternal, birth, and neonatal factors in relation to infants diagnosed with subdural haemorrhage (SDH), by category S06.5 only, category I62.0 only, and SDH and abuse diagnosis combined, and by age 0–6 and 7–365 days during the years 1997–2014 in Sweden. Source population: children born in Sweden: the National Patient Register and the Swedish Medical Birth Register, Swedish National Board of Health and Welfare (*N* = 908,565). ANOVA (means) comparing all and by gender: 1) S06.5 with I62.0, 2) I62.0 with SDH and abuse diagnosis. Mantel-Haenszel Chi-Square or Fisher exact. *P*-value (a <0.001, b<0.01, c <0.05) comparing: 1) all SDH with population, 2) S06.5 with I62.0, 3) I62.0 with SDH and abuse diagnosis.
(DOCX)
