# Supplementary material for: Epidemiology of subdural haemorrhage during infancy: A population-based register study
Source: PLoS One. 2018 Oct 31;13(10):e0206340. doi: 10.1371/journal.pone.0206340 (PMC6209227; doi:10.1371/journal.pone.0206340)
Supplement: S1 Table — Swedish version of 10th revision of the International Statistical Classification of Diseases (ICD-10). (DOCX) [file pone.0206340.s001.docx]

**S1 Table.** Definitions of subdural haemorrhage diagnosis, co-morbidity, neonatal morbidity, and accidents. Swedish version of 10^th^ revision of the International Statistical Classification of Diseases (ICD-10)

|  | **Diagnosis** | **ICD 10 code** |
| --- | --- | --- |
|  |  |  |
| Subdural haemorrhage diagnosis | Subdural haemorrhage due to birth injury | P10.0 |
|  | Subdural haemorrhage acute nontraumatic | I62.0 |
|  | Traumatic subdural haemorrhage | S06.5 |
| Co-morbidity diagnosis | Brain contusion | S060, S061 |
|  | Skull fracture | S020, S021, S028, S0209 S0200, S029 |
|  | Brain oedema | G93.6, S06.1 |
|  | Anoxic brain injury | G93.1 |
|  | Cerebral infarction | I63, I64 |
|  | Sinus venous thrombosis | G08, G95.1, I63.9 |
|  | Hydrocefalus | G91, G94, Q03, Q05 |
|  | Convulsions | R56, R56.8, G40–41, R56.0 |
|  | Retinal haemorrhage | H356 |
|  | Cyanotic attacks of newborn, respiratory failure not elsewhere classified, respiratory arrest | P28.4, J96, R09.2 |
|  | Meningitis | G00, G01, G02, G03 |
|  | Sepsis | A39.2, A40, A41 |
|  | Infantile colic | R10.4 |
|  | Vomiting | R11 |
|  | Gastro-esophageal reflux disease (GERD) | K21.9 |
|  | Rib fracture | S22.3, S 22.4. |
|  | Fracture Long bone | S42.2, S42.3, S42,4, S42.7, S42.8, S52, S72, S82, T10, T12 |
|  | Superficial injury of unspecified body region | T14.0 |
|  | Infant abuse diagnosis (observation for suspected abuse, battered baby syndrome, maltreatment syndrome)  Unspecified event/other specified events, undetermined intent  Homicide | Z03.8K, Y07.9, T74.1, Y06  Y33, Y34  Y09 |
| Pregnancy & delivery complications | Preclampsia | O14, 011 |
|  | Dystocic labour | O62-O66 |
|  |  |  |
| Neonatal morbidity |  |  |
|  |  |  |
|  | Birth injury to scalp | P12 |
|  | Birth injury to skeleton (any fracture) | P13 |
|  | Respiratory distress syndrome (RDS)  Bacterial sepsis of newborn  Other cerebral disturbances of the newborn | P22  P36 |
|  |  | P91 |
|  |  |  |
| Transport accidents |  | V01-99 |
|  |  |  |
| Fall accidents |  | W00-19 |
|  | Fall from the same level | W01 |
|  | Fall while being carried | W04 |
|  | Fall involving bed | W06 |
|  | Fall involving chair or other furniture | W07/W08 |
|  | Fall involving playground equipment | W09 |
|  | Fall on and from stairs and steps | W10 |
|  | Fall on and from ladder | W11 |
