# Supplementary material for: Epidemiology of subdural haemorrhage during infancy: A population-based register study
Source: PLoS One. 2018 Oct 31;13(10):e0206340. doi: 10.1371/journal.pone.0206340 (PMC6209227; doi:10.1371/journal.pone.0206340)
Supplement: S2 Table — Source population: children born in Sweden: the National Patient Register and the Swedish Medical Birth Register, Swedish National Board of Health and Welfare (N = 908,565). ANOVA (means) comparing all and by gender: 1) S06.5 with I62.0, 2) I62.0 with SDH and abuse diagnosis. Mantel-Haenszel Chi-Square or Fisher exact. P-value (a <0.001, b<0.01, c <0.05) comparing: 1) all SDH with population, 2) S06.5 with I62.0, 3) I62.0 with SDH and abuse diagnosis. (DOCX) [file pone.0206340.s002.docx]

**S2 Table.** Mean and median days of SDH diagnosis, distribution of maternal, birth, and neonatal factors in relation to infants diagnosed with subdural haemorrhage (SDH), by category S06.5 only, category I62.0 only, and SDH and abuse diagnosis combined, and by age 0–6 and 7–365 days during the years 1997–2014 in Sweden. Source population: children born in Sweden: the National Patient Register and the Swedish Medical Birth Register, Swedish National Board of Health and Welfare (*N*=908,565). ANOVA (means) comparing all and by gender: 1) S06.5 with I62.0, 2) I62.0 with SDH and abuse diagnosis. Mantel-Haenszel Chi-Square or Fisher exact. *P*-value (a <0.001, b<0.01, c <0.05) comparing: 1) all SDH with population, 2) S06.5 with I62.0, 3) I62.0 with SDH and abuse diagnosis.

|  |  |  | **All SDH^1^** | **SDH**  **0–6 days^2^** | **All**  **7–365 days^3^** | **Only S06.5**  **7–365 days** | **Only I62.0**  **7–365 days** | **SDH & abuse**  **0–365 days^4^** |
| --- | --- | --- | --- | --- | --- | --- | --- | --- |
|  |  |  | **(*n*=306)** | **(*n*=74)** | **(*n*=232)** | **(*n*=108)** | **(*n*=69)** | **(*n*=43)** |
|  |  | ***N*^5^ (%)** | ***n* (%)** | ***n* (%)** | ***n* (%)** | ***n* (%)** | ***n* (%)** | ***n* (%)** |
| **Infant** | |  |  |  |  |  |  |  |
| **Days at diagnosis** | **Mean (all)** |  | 98·3 | 0·88 | 129·3 | 134·2 | 136·6 | 115·2 |
|  | **Mean (females)** |  | 91·5 | 0·79 | 123·3 | 125·1 | 159·2 | 81·5 |
|  | **Mean (males)** |  | 101·9 | 0·93 | 132·5 | 139·7 | 127·4 | 133·2 |
|  | **Median (all)** |  | 73·5 | 0·0 | 105·5 | 100·5 | 124 | 99 |
|  | **Median( females)** |  | 62 | 0 | 101 | 89 | 132 | 78 |
|  | **Median (males)** |  | 77·5 | 0 | 107 | 104 | 107 | 120·5 |
| **Sex** |  |  |  |  |  |  |  |  |
|  | **Female** | 439 067 (48·3) | 108 (35·3) | 28 (37·8) | 80 (34·5) | 41 (38) | 20 (29) | 15 (34·9) |
|  | **Male** | 469 498 (51·7) | 198 (64·7)^a^ | 46 (62·2) | 152 (65·5) | 67 (62) | 49 (71) | 28 (65·1) |
| **Multiple birth** |  | 26 664 (2·9) | 22 (7·2) ^a^ | 4 (5·4) | 18 (7·8) | 6 (5·6) | 7 (10·1) | 4 (9·3) |
| **Maternal characteristics** | |  |  |  |  |  |  |  |
| **Age** | **-34** | 718 814 (79·1) | 254 (83·0) | 60 (81·1) | 194 (83·6) | 88 (81·5) | 57 (82·6) | 40 (93) |
|  | **35+** | 189 751 (20·9) | 52 (17·0) | 14 (18·9) | 38 (16·4) | 20 (18·5) | 12 (17·4) | 3 (7) |
| **Parity** | **Primiparity** | 399 575 (44·0) | 141 (46·1) | 40 (54·1) | 101 (43·5) | 48 (44·4) | 27 (39·1) | 22 (51·2) |
|  | **Multiparity** | 508 990 (56·0) | 165 (53·9) | 34 (45·9) | 131 (56·5) | 60 (55·6) | 42 (60·9) | 21 (48·8) |
| **Preeclampsia** |  | 38 833 (4·3) | 27 (8·8) ^a^ | 7 (9·5) | 20 (8·6) | 5 (4·6) | 6 (8·7) | 6 (14·0) |
| **Dystocic labour** |  | 107 254 (11·8) | 58 (18·9) ^a^ | 24 (32·4) | 34 (14·7) | 20 (18·5) | 6 (8·7) | 7 (16·3) |
| **Mode of delivery** | **Caesarean planned** | 82 150 (9·0) | 39 (12·7)^c^ | 2 (2·7) | 37 (15·9) | 10 (9·3) | 11 (15·9) | 8 (18·6) |
|  | **Normal vaginal delivery** | 491 627 (54·1) | 132 (43·1) ^a^ | 20 (27) | 112 (48·3) | 60 (55·6) | 32 (46·4) | 19 (44·2) |
|  | **Not normal spontaneous vaginal delivery** | 190 697 (21) | 62 (20·3) | 14 (18·9) | 48 (20·7) | 21 (19·4) | 16 (23·2) | 9 (20·9) |
|  | **Emergency caesarean** | 78 650 (8·7) | 30 (9·8) | 8 (10·8) | 22 (9·5) | 9 (8·3) | 8 (11·6) | 5 (11·6) |
|  | **Assisted vaginal delivery** | 65 447 (7·2) | 43 (14·1) ^a^ | 30 (40·5) | 13 (5·6) | 8 (7·4) | 2 (2·9) | 2 (4·7) |
| **Neonatal characteristics** | |  |  |  |  |  |  |  |
| **Gestational week** | **37+** | 851 572 (93·8) | 263 (85·7) | 67 (90·5) | 196 (84·5) | 95(88) | 58 (84·1) | 35 (81·4) |
|  | **32-36** | 47 412 (5·2) | 32 (10·8) ^a^ | 4 (5·4) | 29 (12·5) | 11 (10·2) | 9 (13·0) | 5 (11·6) |
|  | **<32** | 9 146 (1·0) | 10 (3·3) ^a^ | 3 (4·1) | 7 (3·0) | 2 (1·9) | 2 (2·9) | 3 (7) |
| **Small-for-gestational-age** | **<2.5th percentile** | 20 574 (2·3) | 15 (5·3) ^a^ | 3 (4·1) | 12 (5·2) | 3 (2·8) | 4 (6·6) | 5 (11·6) |
|  | **<10th percentile** | 94 275 (10.4) | 40 (13.3) ^a^ | 7 (9.5) | 33 (14.2) | 14 (13.0) | 11 (16.4) | 7 (16.3) |
| **Birth Asphyxia** | **Apgar <4 1 minute** | 12 149 (1·3) | 21 (6·9) ^a^ | 16 (21·6) | 5 (2·2) |  | 1 (1·4) | 0 |
|  | **Apgar <4 5 minutes** | 2 343 (0·3) | 6 (2.0) ^a^ | 6 (8.1) | 0 | 0 | 0 | 0 |
|  | **Apgar <4 10 minutes** | 1 306 (0.14) | 5 (1·6) ^a^ | 5 (6·8) | 0 | 0 | 0 | 0 |
| **Neonatal diagnosis** | **Birth injury to the scalp** | 9 164 (1·0) | 21 (6·9) ^a^ | 14 (18·9) | 7 (3·0) | 3 (2·8) | 2 (2·9) | 0 |
|  | **Birth injury to the skeleton** | 5 051 (0·56) | 8 (2·6) ^a^ | 8 (10·8) | 0 | 0 | 0 | 0 |
|  | **Respiratory distress** | 34 120 (3·8) | 25 (8·2) ^a^ | 9 (12·2) | 16 (6·9) | 7 (6·5) | 4 (5·8) | 3 (7) |
|  | **Sepsis** | 9 235 (1·0) | 10 (3·3) ^a^ | 3 (4·1) | 7 (3·0) | 3 (2·8) | 2 (2·9) | 1 (2·3) |
|  | **Convulsions** | 2 436 (0·27) | 38 (12·4) ^a^ | 31 (41·9) | 7 (3·0) | 1 (0·9) |  | 1 (2·3) |
|  | **Other cerebral disturbances of the newborn** | 686 (0·08) | 6 (2·0) ^a^ | 6 (8·1) | 0 | 0 | 0 | 0 |

^1^55 cases with P10.0 diagnosis, ^2^48 cases with P10.0 diagnosis, ^3^7 cases with P10.0 diagnosis, ^4^1 case day 1, ^5^Source population=908,565
